# AChR antibodies show a complex interaction with human skeletal muscle cells in a transcriptomic study

**DOI:** 10.1038/s41598-020-68185-x

**Published:** 2020-07-08

**Authors:** Yu Hong, Xiao Liang, Nils Erik Gilhus

**Affiliations:** 1 Department of Clinical Medicine, University of Bergen, Bergen, Norway; 2 Department of Neurology, Haukeland University Hospital, Bergen, Norway

**Keywords:** Neuroimmunology, Gene regulatory networks, Neuromuscular disease

## Abstract

Acetylcholine receptor (AChR) antibodies are the most important pathogenic marker in patients with myasthenia gravis (MG). The antibodies bind to AChRs on the postsynaptic membrane, and this leads to receptor degradation, destruction, or functional blocking with impaired signal at the neuromuscular junction. In this study, we have explored the effects of AChR antibodies binding to mature human myotubes with agrin-induced AChR clusters and pathways relevant for AChR degradation using bulk RNA sequencing. Protein-coding RNAs and lncRNAs were examined by RNA sequencing analysis. AChR antibodies induced marked changes of the transcriptomic profiles, with over 400 genes differentially expressed. Cholesterol metabolic processes and extracellular matrix organization gene sets were influenced and represent AChR-trafficking related pathways. Muscle contraction and cellular homeostasis gene sets were also affected, and independently of AChR trafficking. Furthermore, we found changes in a protein-coding RNA and lncRNA network, where expression of lncRNA MEG3 correlated closely with protein-coding genes for cellular homeostasis. We conclude that AChR antibodies induce an active response in human skeletal muscle cells which affects key intra- and extracellular pathways.

## Introduction

AChR antibodies represent the main pathogenic marker of the autoimmune disease myasthenia gravis^1^. At the neuromuscular junction (NMJ), AChR antibodies exert their effect by cross-linking AChR receptors in the muscle cell membrane, and this leads to degradation through antigenic modulation^2^. The antibodies also bind complement and induce complement-mediated destruction of the AChR receptors and the membrane structure. AChR can in addition be blocked by direct antibody-receptor binding^1^. The main immune region (MIR) for AChR antibody binding locates at the α subunit of both adult type and fetal type AChR^3–5^.

The maintenance and stability of AChRs at the NMJs is a balanced, dynamic process^6^. Agrin released from the presynaptic membrane aggregates AChRs through agrin-LRP4-MuSK pathways^7^. Cholesterol and extracellular matrix also play roles in stabilizing AChR clusters^8,9^. The AChR antibodies from MG patients bind to AChRs and break this balance. It has been reported that AChR internalization can be triggered by both AChR antibodies and α-bungarotoxin binding with quantum dots^10,11^. One study found that clathrin and caveolin were both required for endocytosis of AChRs in Xenopus primary muscle cells^10^. Another study found that AChRs were internalized via an endocytic pathway in C2C12 and Chinese hamster ovary cell lines, distinct from the clathrin-mediated caveolar pathway^11^. Cytoskeleton structures are required for AChR internalization after the binding of AChR antibodies^10,11^.

Current knowledge regarding AChR internalization is largely based on animal cell lines expressing AChRs and focused on a few AChR relevant pathways. The mechanisms of antibody-induced AChR internalization in human skeletal muscle cells have not been studied in detail. It is unclear whether the AChR antibodies influence additional skeletal muscle physiological functions after disrupting AChRs.

Elucidating the effect of AChR antibodies on human skeletal muscle cells using transcriptomic methods should help to reveal myasthenia gravis pathogenesis and to identify potential treatment targets. We have used human myotubes with mature sarcomere structures that express AChR clusters to explore their transcriptomic status after incubation with AChR antibodies by using RNA sequencing. The antibodies induced changes in the transcriptome expression, involving both AChR trafficking and muscle physiology pathways. We identified a co-expression network including lncRNA MEG3, playing a role in cellular homeostasis maintenance, that was selectively deregulated by the AChR antibodies.

## Results

### AChR antibodies bind to AChRs on human myotubes

**Figure 1:**
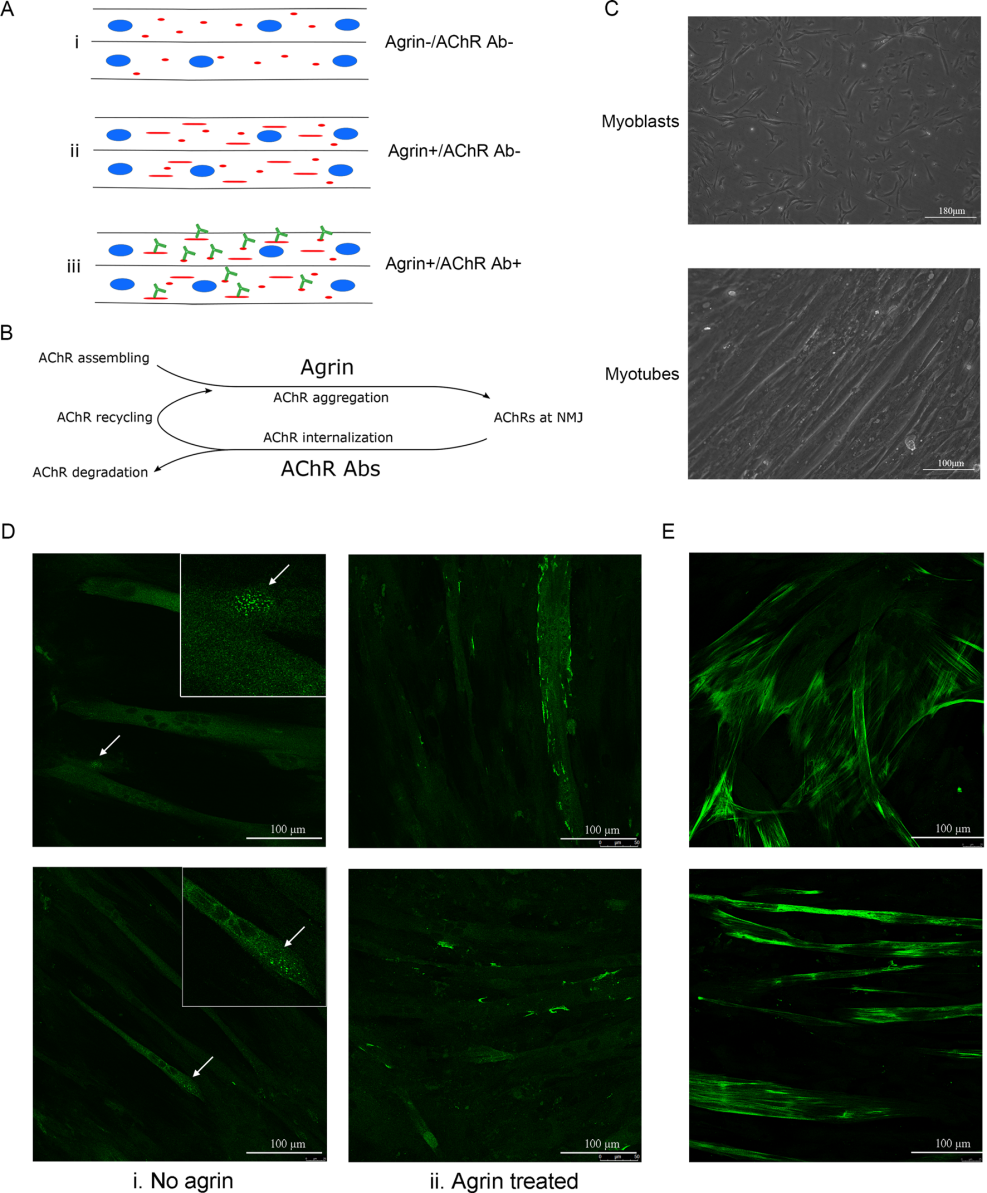
Skeletal muscle cell differentiation and AChR antibody binding to AChRs. (**A**) Three groups (Agrin−/Ab−, Agrin+/Ab− and Agrin+/Ab+) of differentiated skeletal muscle cells were examined. (**B**) Agrin and AChR antibodies affects AChR dynamics. (**C**) Differentiated and undifferentiated skeletal muscle cells in culture. (**D**) AChR antibodies bound to both clustered (agrin-treated) and unclustered (no agrin) AChRs on myotubes. AChRs were stained with the AChR antibody (mAb198). (**E**) Mature myotubes expressed myosin proteins.

To establish a cell model that expressed AChRs (Fig. 1A,B), human primary myoblast cells were differentiated into myotubes (Fig. 1C). Mature myotubes were observed from day 4–5 after initiating the differentiation (Fig. 1C). The differentiation efficiency of myotubes reached 70% at differentiation day 5 (Fig. S1). Spontaneous, unclustered AChRs were formed on the myotubes (Fig. 1D-i). Mature myotubes had sarcomere structure and expressed myosin filament (Fig. 1E).

AChRs on the postsynaptic membrane are clustered and stabilized by agrin released from the axon terminal at the NMJ in vivo^7^. In our study, to induce AChR clusters, recombinant mice agrin (300 ng/ml) was added to skeletal muscle cells from differentiation day 1–2, and typical AChR clusters were visualized after 1–2 days (Fig. 1D-ii). To explore the effect of AChR antibodies on myotubes with AChR clusters, the recombinant monoclonal AChR antibody mAb198, that targets the main immune region of AChR, was added overnight (16 h) to agrin-treated myotubes at day 4–6. Previous studies have shown that mAb198 is able to crosslink adjacent AChRs and induce AChR internalization^2^. In our system, mAb198 bound to both unclustered and clustered AChRs (Fig. 1D).

### AChR antibodies induced marked changes in transcriptomic profiles

To explore the effect of the AChR antibody on mature human myotubes, bulk RNA sequencing was conducted on three groups of skeletal muscle cells; Agrin−/Ab−, Agrin+/Ab−, and Agrin+/Ab+ (Fig. 1A). Four biological replicates were included for each group. The polyA enrichment method was applied when building RNA libraries. In average, 40 million paired-end reads were generated from each sample. A standard RNA sequencing data analysis workflow was applied (Fig. S2). The adapters were trimmed. Raw sequencing data was quality checked, and the reads with low quality were removed. Clean reads were then aligned to the reference genome (GRCh38), which has included more than 60,000 protein-coding, pseudo, and non-coding genes. Reads that were mapped to specific genes were counted.

**Figure 2:**
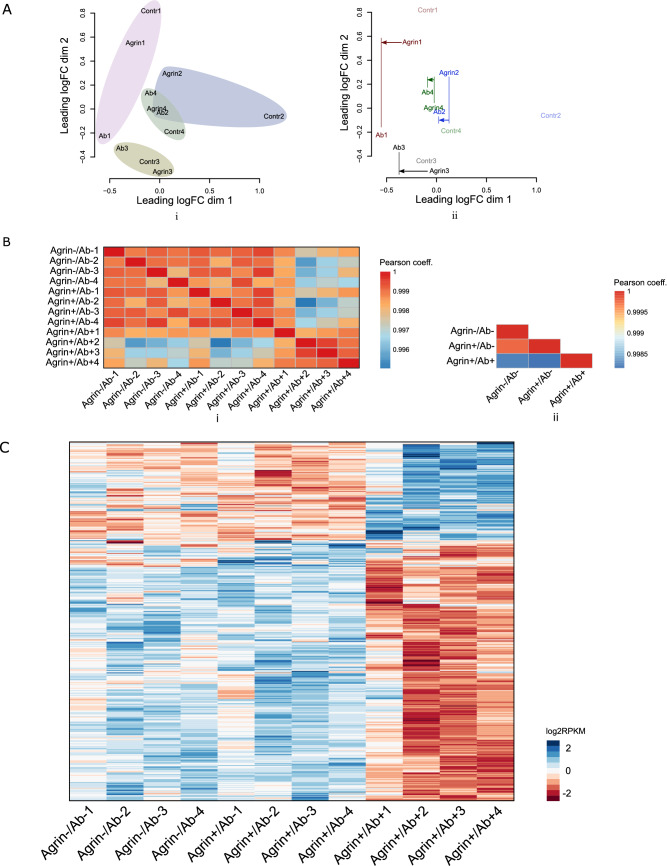
Transcriptome profiles of differentiated skeletal muscle cells and the effect of AChR antibodies at widespread level. (**A**) The principle component analysis showed both a batch effect (i) and an AChR antibody treatment effect (ii). Within each batch, the antibody treated samples shift to the left for the first component (*x*-axis) compared with non-antibody treated groups (ii). *Contr*: Agrin−/Ab−; *Agrin*: Agrin+/Ab−; *Ab*: Agrin+/Ab+. (**B**) Transcriptome similarities between samples (i) and groups (ii) were calculated based on Pearson correlation coefficient. Incubation with AChR antibodies gave marked changes in the transcriptomic profiles. (**C**) The heatmap showed the DE genes between Agrin+/Ab− and Agrin+/Ab+ groups.

To observe the AChR antibody-induced effect on transcriptomic profiles of skeletal muscle cells at a widespread level, we first constructed a multidimensional scaling plot based on principle component analysis (Fig. 2A). This plot showed both a batch effect (Fig. 2A-i) and an AChR antibody treatment effect (Fig. 2A-ii). Then we measured the sample and group similarities by calculating the Pearson correlation coefficients between all samples and between all groups for the whole RNA expression dataset (Fig. 2B). The AChR antibodies induced marked changes in the transcriptomic profile of the skeletal muscle cells at both sample (Fig. 2B-i) and group level (Fig. 2B-ii).

To determine the genes that were differentially expressed (DE) by the AChR antibody, we conducted differential expression analysis. A generalized linear model considering the batch effect was used. After adjusting for batch effects, we found that 410 protein-coding RNAs, 20 pseudogene RNAs, 3 antisense RNAs, and 9 lncRNAs were expressed differently in the Agrin+/Ab+ group compared with the Agrin+/Ab− group (Padj < 0.05) (Fig. 2C). Table S1 lists the 30 protein-coding RNAs that showed the biggest difference in expression with and without AChR antibodies. Table S2 lists similar information for the 9 DE lncRNAs and the 3 antisense RNAs.

### DE protein-coding RNAs were enriched in distinct functional gene sets

**Figure 3:**
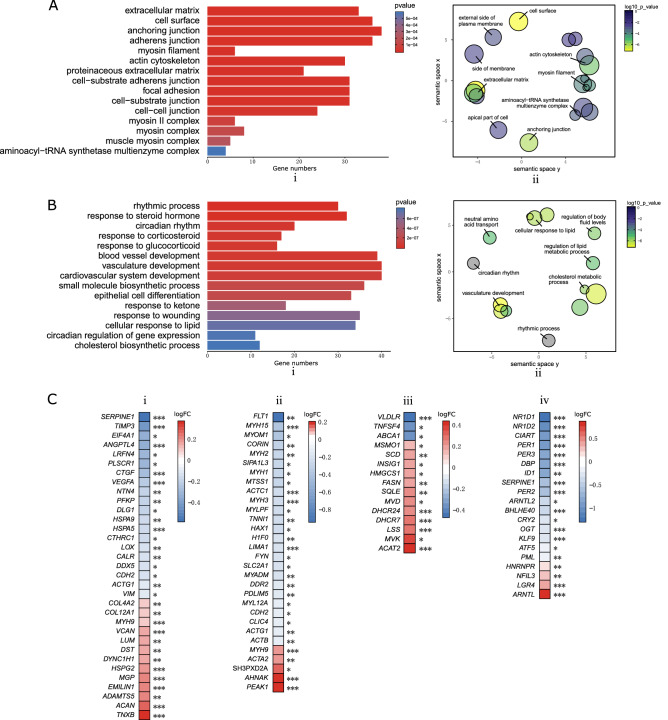
Gene set enrichment analysis of DE genes. (**A**) 29 significantly enriched gene sets for cellular components were identified. The top 15 sets are listed (i) and all 29 illustrated by a semantic similarity-based scatter plot (ii). Enriched gene sets included extracellular matrix, actin cytoskeleton and the myosin gene family. (**B**) 225 significantly enriched gene sets for biological processes were identified. The top 15 sets are listed (i) and the top 30 illustrated by a semantic similarity-based scatter plot (ii). Enriched gene sets included rhythmic processes, and cholesterol and lipid metabolic processes. (**C**) DE genes reflecting extracellular matrix (i), actin cytoskeleton (ii), cholesterol metabolic processes (iii), and circadian rhythms (iv). ***Padj < 0.001, **Padj < 0.01, *Padj < 0.05.

To explore if the DE protein-coding RNAs were enriched in functional gene groups, we conducted gene ontology enrichment analysis, which implemented a hypergeometric model to examine whether the number of DE genes belonging to one functional gene set was higher than expected. The gene ontology analysis showed that DE genes were enriched in pathways regulating the extracellular matrix, actin cytoskeleton, and myosin filament (Fig. 3A-i,ii), and also for genes regulating cholesterol metabolic processes and circadian rhythms (Fig. 3B-i,ii). Several genes encoding extracellular matrix proteoglycans, including collagen (COL12A1, COL4A2), aggrecan (ACAN) and versican (VCAN), showed an increased expression after AChR antibody incubation (Fig. 3C-i). The DE actin family genes included ACTB, ACTA2, ACTC1, and ACTG1, and the myosin family genes included MYH1, MYH2, MYH3, MYH9, MYH15, MYL12A, MYLPF (Fig. 3C-ii). Regarding the cholesterol metabolic process, the gene VLDLR was down-regulated, whereas DHCR24, DHCR7 and LSS were up-regulated (Fig. 3C-iii). Also, a cholesterol homeostasis-related protein, LRP1, was up-regulated after AChR antibody treatment (Table S1). Several key regulators of circadian rhythms including Per (PER1, PER3) and Rev-erba (NR1D1, NR1D2), together with MET and CXCL1 which regulate cell responses to external stimuli, were differentially expressed (Fig. 3C-iv).

### Time series analysis reveals expression patterns of protein-coding RNAs

**Figure 4:**
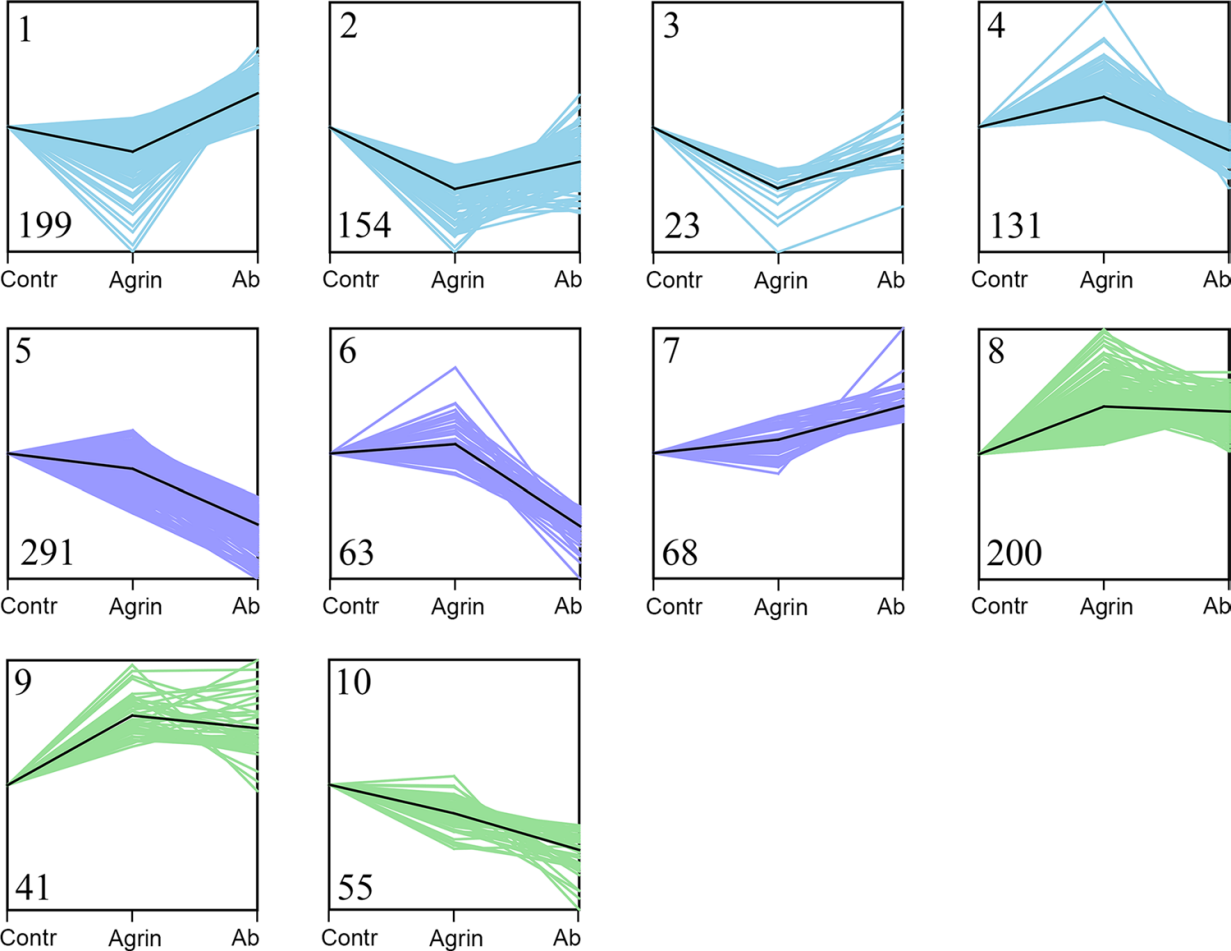
Short time series analysis in three consecutive groups of differentiated skeletal muscle cells. *Contr*: Agrin−/Ab−; *Agrin*: Agrin+/Ab−; *Ab*: Agrin+/Ab+. Ten clusters were generated using the K-means clustering algorithm based on the consecutive expression pattern of group Agrin−/Ab−, Agrin+/Ab−, and Agrin+/Ab+. Cluster 1–4 (in light blue) had opposite trends in group Agrin+/Ab− (versus Agrin−/Ab−) and group Agrin+/Ab+ (versus Agrin+/Ab−), and were classified as AChR trafficking related clusters; Cluster 5–7 (in violet) had a deviated trend only in Agrin−/Ab− group when compared to the other two groups, and were classified as muscle physiology pathway associated clusters. Cluster 8–10 (in green) were the remaining clusters without a distinct relation to AChR antibodies.

Having identified the DE genes and functional gene clusters affected by the AChR antibody, we explored the genes being specifically involved in the AChR trafficking process. First, we examined the three biological consecutive groups: Agrin−/Ab−, Agrin+/Ab−, and Agrin+/Ab+ (Fig. 1A), and conducted a short time series analysis for all the protein-coding RNAs (Fig. 4)^12^. The K-means clustering algorithm was applied to cluster the genes with a similar expression pattern across the three groups into one cluster^12^. In total ten clusters were characterized. The agrin at NMJ exerts its effect on the AChR synthesis pathway, while AChR antibodies act on the AChR degradation pathway (Fig. 1B). Clusters with opposite trends in the Agrin+/Ab− (versus Agrin−/Ab−) and the Agrin+/Ab+ (versus Agrin+/Ab−) groups were classified as AChR trafficking related (Cluster 1–4) (Fig. 4). Clusters with a deviating trend only in the Agrin−/Ab− group were classified as muscle physiology associated (Cluster 5–7) (Fig. 4).

### AChR antibodies induced changes in AChR trafficking and muscle physiology related pathways

**Figure 5:**
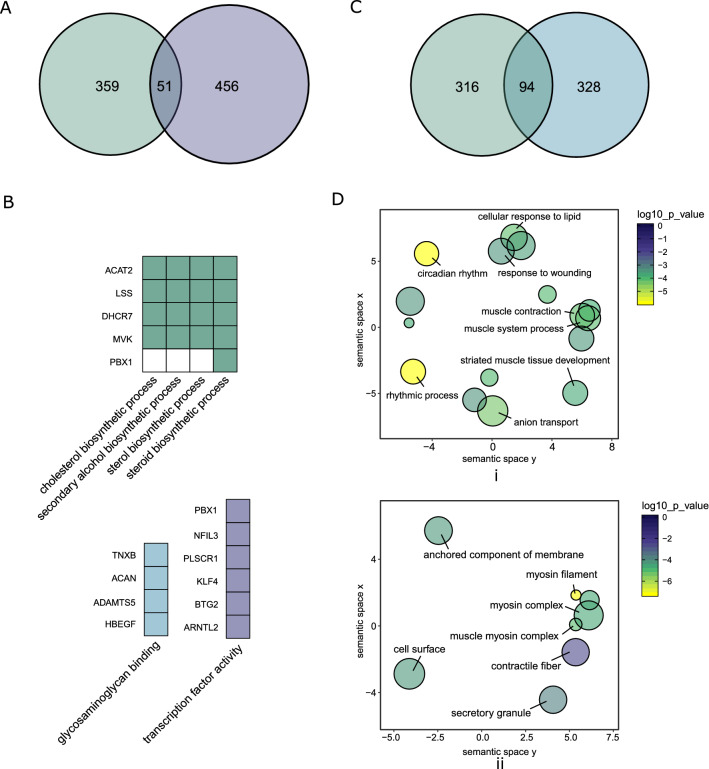
AChR antibodies induced changes of AChR trafficking and muscle physiology related pathways. (**A, B**) 51 genes were identified by intersecting between the AChR trafficking related gene clusters and the DE protein-coding genes (**A**). They were enriched for cholesterol metabolic processes, and glycosaminoglycan binding and transcription factor activity (**B**). (**C**,**D**) 94 genes were identified by intersecting between the muscle physiology associated clusters and DE protein-coding genes (**C**). They were enriched for cellular homeostasis (rhythmic process, cellular response to wound and lipid, etc.) (**D**-i) and muscle contraction related pathways (**D**-i,ii).

We identified which genes from the AChR trafficking related gene clusters (Cluster 1–4) and from the unrelated clusters (Cluster 5–7) were affected by the AChR antibody. We performed intersections between the 410 DE protein-coding genes and each of the two cluster groups (Fig. 5). We found 51 DE genes in the AChR trafficking related clusters (Fig. 5A). They were enriched in gene sets of cholesterol metabolic processes (ACTA2, LSS, DHCR7, MVK, PBX1), glycosaminoglycan binding (TNXB, ACAN, ADAMTS5, HBEGF) and transcription factor activity (NFIL3, PLSCR1, KLF4.) (Fig. 5B). 94 DE genes were identified in muscle physiology pathway associated clusters (Fig. 5C). Genes for cellular homeostasis related pathways were enriched (rhythmic process, cellular response to wound and lipid.) (Fig. 5D-i), and also muscle contraction genes (muscle contraction, myosin filament, contractile fiber.) (Fig. 5D-i,ii).

### LncRNA and protein-coding RNA co-expression networks showed an association between lncRNA MEG3 and cellular homeostasis

**Figure 6:**
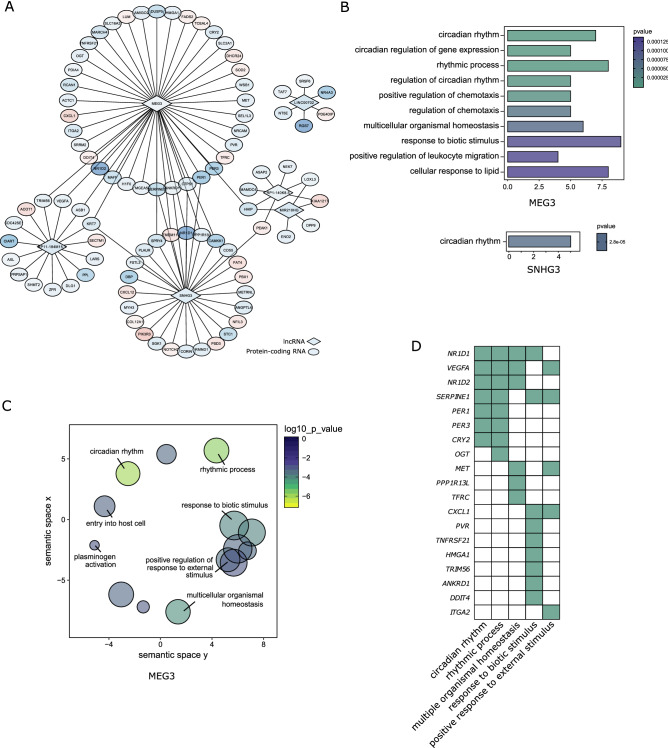
LncRNA and protein-coding RNA co-expression network. (**A**) A co-expression network was built based on the correlation efficient between all DE protein-coding RNAs and lncRNAs. LncRNA MEG3, SNHG3, and RP11-184M15.1 were correlated with several protein-coding RNA groups. (**B**,**C**) Gene set enrichment analysis of lncRNA correlated with protein-coding RNAs. 29 enriched gene sets for MEG3 correlated protein-coding RNAs, and one gene set (circadian rhythm) for SNHG3 correlated protein-coding RNAs were identified (**B**). The 29 enriched gene sets for MEG3 correlated protein-coding mRNAs were illustrated by a similarity-based scatter plot (**C**). The enriched gene sets for lncRNA MEG3 included circadian rhythm, response to biotic stimuli, and positive response to external stimuli. (**D**) 19 out of 46 MEG3 correlated protein-coding RNAs belonged to cellular homeostasis-related gene sets.

Long noncoding RNAs (lncRNAs) are extensively involved in biological regulation^13,14^, and they are more species- and tissue-specific than protein-coding RNAs^15^. 9 DE lncRNAs were identified in the Agrin+/Ab+ group compared with the Agrin+/Ab− group. In order to explore the roles of the DE lncRNAs and their regulatory targets in the presence of AChR antibodies, a lncRNA and protein-coding RNA co-expression network was constructed based on the Pearson correlation efficient. The 410 DE mRNAs and 9 DE lncRNAs were included. mRNA-lncRNA pairs with coefficient > 0.9 (positive correlation) or < − 0.9 (negative correlation) were defined as correlated pairs. We found that the lncRNAs MEG3, RP11-184M15.1, and SNHG3 were co-expressed with several protein-coding RNA groups (Fig. 6A). MEG3 in particular showed an association with intracellular homeostasis related pathways, affecting the protein-coding gene sets of circadian rhythms, multicellular homeostasis and response to external stimuli (Fig. 6B–D).

### Validation of RNA sequencing using Real-Time quantitative PCR (RT-qPCR)

**Figure 7:**
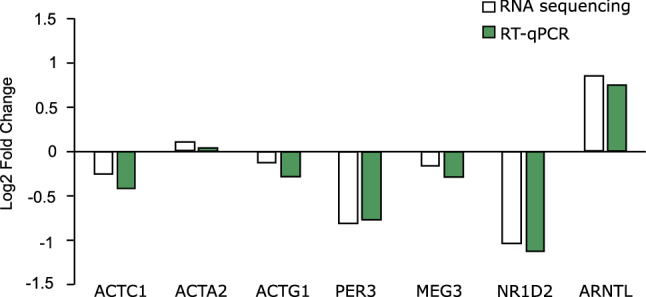
RT-qPCR validation for RNA sequencing results. Six genes (ACTA2, ACTC, ACTG1, ARNTL, PER3, NR1D2) from relevant pathways (actin cytoskeleton, circadian rhythm), and one lncRNA (MEG3) were validated.

Six genes (ACTA2, ACTC, ACTG1, ARNTL, PER3, NR1D2) from relevant pathways and one lncRNA (MEG3) were chosen for validation of the RNA sequencing results by using RT-qPCR. RT-qPCR was conducted using the same RNA samples as for the sequencing. RT-qPCR and RNA sequencing showed the same trend for all seven genes (Fig. 7, Fig. S4).

## Discussion

We have found that treatment of well-developed human skeletal muscle cells in culture with AChR antibodies leads to extensive changes in the expression of both protein-coding RNAs and lncRNAs. More than 400 genes were differentially expressed after AChR antibody incubation. Those genes were enriched in distinct functional groups including formation and maintenance of both AChR-trafficking dependent and independent processes. Pathways relating to the extracellular matrix, regulation of cholesterol metabolism, actin and myosin gene family, and intracellular homeostasis related pathways were involved. Furthermore, lncRNAs that regulate intracellular homeostasis showed a change in expression.

**Figure 8:**
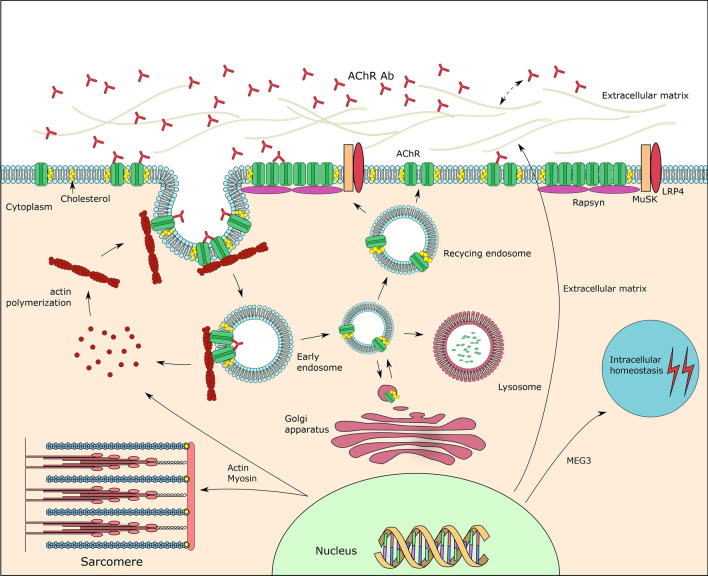
AChR antibody-induced effects on myotubes expressing AChR clusters. 1. Cholesterol is involved in AChR internalization, modulation and recycling. 2. The dynamic process of actin polymerization and turnover is involved in AChR internalization. 3. Multiple myosin family genes and muscle contraction pathway genes were differentially expressed. 4. AChR antibodies induced an active response of extracellular matrix molecules. 5. Dysregulation of cellular homeostasis genes correlated closely with changes in lncRNA MEG3 gene expression.

Multiple genes involved in the cholesterol metabolic process were up- or down- regulated. Cholesterol is an important component of the lipid bilayer cell membrane structure. Cholesterol influences a variety of AChR-related biological processes including AChR synthesis, endocytosis, recycling, and AChR cluster stability (Fig. 8)^8^. Cholesterol is an essential partner of AChR both on the cell membrane and for intracellular AChR trafficking^8,16^. The AChR trafficking process needs cholesterol already in the endoplasmic reticulum and Golgi apparatus^8^. A cholesterol recognition motif in the transmembrane domain of several AChR subunits has been identified^17,18^. Depletion of cholesterol will promote AChR degradation^19^. Our study illustrates and supports the importance of cholesterol for AChR and AChR function. Sphingolipids, another component of the lipid raft on the post-synaptic membrane, are also associated with AChR trafficking^20^. One study showed that genes regulating biosynthesis of sphingolipids were dysregulated in cultured cells from patients with MG and AChR antibodies^21^. Furthermore, we found a cholesterol metabolism related protein, LRP1, to be up-regulated after AChR antibody treatment. LRP1 is involved in several biological processes that maintain cholesterol homeostasis^22,23^. LRP1 is also a membrane vesicle-trafficking protein. LRP1 can bind more than 40 extracellular ligands and membrane receptors, and the molecule helps transporting its ligands into the cytoplasm^24^. LRP1 could act as a cargo protein for AChR during AChR internalization, or alternatively regulate AChR by influencing cholesterol metabolism.

The changes in RNA expression induced by the AChR antibody influence extracellular matrix molecules. AChR autoantibodies bind to the extracellular part of the receptor^1^. Extracellular matrix is important for tissue repair and regeneration^25,26^, and its glycoproteins constitute important components of the NMJ^27^. Specific collagen and laminin protein isoforms play important roles in maintaining the topological structure of the NMJ and regulating AChR maturation^9^. In our study, several extracellular matrix components including collagen IV, aggrecan, and versican were up-regulated by the AChR antibody. Further, our analysis showed that the glycosaminoglycan binding gene cluster was linked to AChR trafficking processes. Previous studies of both MG patients and animals have showed a dysregulation of extracellular matrix molecules in the skeletal muscle cells of patients with MG and in experimental autoimmune myasthenia gravis (EAMG) models^28,29^. Interactions between extracellular matrix organization, AChR trafficking, and AChR antibodies need further studies.

Multiple actin family genes were up- or down-regulated by the AChR antibody incubation. The internalization of AChR vesicles is in part controlled by the intracellular cytoskeleton^10,11^. The dynamic process of actin polymerization and turnover is actively involved in internalization of AChR and other membrane receptors (Fig. 8)^11,16,30^. Since the membrane receptor internalization requires actin-assembling dynamics, AChR internalization might inversely influence actin gene expression. One hypothesis is that AChR internalization and endocytosis needs a fast cycling of actin polymerization and turnover. This should lead to higher levels of non-polymerized actin, which should again inhibit cytoskeleton gene expression. Another possibility is that the intracellular endosome transport needs a less dense environment. A high cytoskeleton density should inhibit the endosome movement. A previous study found in fact significantly enriched gene sets of actin cytoskeleton and actin filament in EAMG rats compared with controls in an RNA microarray study^28^.

Our study showed that an AChR antibody induced changes of muscle contraction related pathways, including the muscle myosin complex and myosin filament gene sets. Myosin family genes are key components of the sarcomere (Fig. 8) and are expressed in mature myotubes (Fig. 1). Instead of being directly linked to the AChR trafficking process, our data indicated that such effects seemed to be mediated by alternative pathways or were indirect consequences of the AChR internalization process. As both muscle contraction and cellular homeostasis related pathways had similar expression patterns in our model, we hypothesis that muscle contraction is influenced by the breakdown of cellular homeostasis pathways induced by AChR antibodies. Several studies have shown that myosin and muscle contraction related genes are influenced by AChR antibodies in muscle cell cultures or in patients^28,31,32^. Maurer et al. showed that myofibril and sarcomere associated genes were dysregulated in MG patient and EAMG rat muscle^28^. Another study found that myosin II related genes were differentially expressed in cultured muscle cells from MG patients compared with healthy controls^31^. The influence of AChR antibodies on muscle contraction related genes may have pathophysiological and clinical consequences.

Several lncRNAs were differentially expressed in the presence of AChR antibodies. We have built a protein-coding RNA and lncRNA cooperation network. The lncRNA MEG3 correlated closely with intracellular homeostasis gene groups including genes important for circadian rhythms, multicellular homeostasis and responses to external stimuli. MEG3 is best known as a tumor suppressor gene that activates p53^33,34^. MEG3 seems to play a role in maintaining intracellular homeostasis in skeletal muscle cells, and its down-regulation in the presence of AChR antibodies may have functional consequences. Previous studies in EAMG rats have shown that gene groups controlling responses to stress and stimuli were dysregulated in their skeletal muscles^29,32^.

AChR antibodies occur in vivo in myasthenia gravis patients where they induce muscle weakness. We have found that AChR antibodies induce an active response in cultured human skeletal muscle cells. An important limitation of our study is that it is an in vitro study of human cultured cells with a purified AChR antibody against the MIR of the AChR α subunit. Another limitation is that not all the cultured myoblasts differentiated into myotubes. We have optimized our differentiation conditions to reach a differentiation efficiency of around 70%. The expression of AChRs was most dense during the early stages of cell differentiation (during the first 7 days).

In summary, we have found that the well-known process of AChR internalization in the presence of an AChR antibody was accompanied by dysregulation of genes controlling cholesterol metabolism and extracellular matrix. Muscle contraction and cellular homeostasis gene sets being independent of AChR trafficking were also dysregulated. The effects observed by us in vitro may contribute to the clinical picture of myasthenia gravis and point to potential treatment targets.

## Materials and methods

### Cell culture

Human primary myoblast cells were purchased from a commercial source (Cook Myosite, US) and had been isolated from a female, healthy donor aged 32 years. The myoblasts were cultured with serum free growth medium (Cook Myosite) with 20% fetal bovine serum (Gibco, Thermo Fisher Scientific, US). When the cells had grown to 90–95% confluence, they were cultured with differentiation medium. Differentiation medium was composed of Myotonic serum free differentiation medium (Cook Myosite), 2% horse serum (Gibco) and 1% Insulin-Transferrin-Selenium (Thermo Fisher Scientific). 300 ng/ml agrin was added to the cultures 1–2 days after the switch to differentiation medium. 10 nM recombinant human AChR IgG antibodies (mAb198) (Absolute antibody, UK) were added to the culture medium at differentiation day 4–6, and incubated with the cell cultures for 16 h. Parallel control cultures without AChR antibodies were also examined. mAb198 is a highly purified IgG protein. All the antibodies have the same protein sequence, which includes an antigen binding site against MIR of the AChR α-subunit. Therefore, in order to avoid introducing additional batch effect when conducting high-throughput transcriptomic sequencing, control IgG was not included in the control groups without AChR antibodies.

### Immunofluorescence

For staining with mAb198, cells grown on coverslips were fixed with 4% paraformaldehyde diluted in phosphate-buffered saline (PBS) for 10 min. Cells were blocked using blocking buffer (PBS with 0.1% Tween, 1% BSA and 22.52 mg/ml Glycine) for 30 min at room temperature. mAb198 diluted 1:200 in blocking buffer were added to the cells and incubated overnight at 4 °C. After washing three times with PBS, the cells were incubated with Alexa fluor 488 goat anti-human IgG antibodies (Invitrogen, Thermo Fisher Scientific) diluted 1:400 for 50 min at room temperature. After three washings with PBS, cells were stained with ProLong Diamond Antifade Mountant with DAPI (Invitrogen). Coverslips with the cells were mounted to microslide, before observation under a Leica TCS SP5 Confocal microscope (Leica, Germany).

### RNA sequence

Four replicates of AChR antibody-treated and untreated myotubes were included for RNA sequencing. Each pair of cell samples (AChR antibody-treated versus control) was seeded from the same passaging cells. Cells were tested negative for mycoplasma contamination before RNA isolation. Total RNA was isolated from mature myotubes using RNeasy Mini Kit (QIAGEN). RNA quality and concentration were checked using Bioanalyzer and Qubit. RNA-seq libraries were established by the polyA enrichment method^35^. The sequencing was conducted by StarSEQ (Mainz, Germany) using a Illumina Next Seq 500 platform. In average 40 million paired end reads (150 bp) were generated from each RNA sample. The workflow of the analysis process is shown in Fig. S2. Adapter was trimmed using Trimmomatic (version 0.38)^36^. Reads were aligned to human genome (GRCh38.84, Ensemble) using HISAT2^37^.

### Differential expression analysis

Read counts were generated using HTSeq-count^38^. Differential expression analysis was conducted using the EdgeR (R package)^39^. A generalized linear model considering the batch effect was used for the analysis. A design matrix was applied considering both grouping (antibody versus no antibody) and batch effects (pairs of samples) to fit the model. The Cox-Reid profile-adjusted likelihood method was used to estimate dispersion^39^. Quasi-likelihood F-tests were used to calculate which genes’ expression differed significantly. In EdgeR, counts per million reads (CPM) was used as measurement of RNA expression level, and fold change (FC) (log2 transformed) was used to determine the up- (> 0) or down-regulation (< 0) of RNA levels in the treatment group versus controls. We adjusted for multiple comparisons by using the false discovery rate method. Only genes with adjusted P value < 0.05 were considered as significantly differentially expressed^39^. Since we had well-controlled baseline conditions and the same myotube differentiation stage in all groups, FCs were not filtered for DE genes.

### Principle component and gene ontology enrichment analysis

Principle component analysis was conducted using EdgeR^39^. A multidimensional scaling plot was used to show inter-group differences and batch effects of samples. Gene ontology analysis was conducted using ClusterProfiler (R package) to cluster the significantly differentially expressed genes based on biological processes and cellular components, using the over-representation test^40^. This statistic test implements a hypergeometric model to examine whether the number of selected genes under certain conditions is larger than expected^41^. The *P* value was calculated based on the following formula:

$$p = 1 - \mathop \sum \limits_{i = 0}^{k - 1} \frac{{\left( {\begin{array}{*{20}c} M \\ i \\ \end{array} } \right)\left( {\begin{array}{*{20}c} {N - M} \\ {n - i} \\ \end{array} } \right)}}{{\left( {\begin{array}{*{20}c} N \\ n \\ \end{array} } \right)}}$$

where N refers to total number of background genes; M refers to the number of genes in one specific gene set within this background; n refers to the number of DE genes; k refers to the number of genes in this specific gene set that were DE genes. P value < 0.05 was considered statistically significance. Gene ontology lists were further visualized using semantic similarity-based scatter plots^42^.

### Short time series analysis

Short time series analysis was conducted using STEM^12^. Protein-coding RNAs with reads per kilobase of exon per million reads mapped (RPKM) > 0.1 were included in the analysis. RPKM is a unit to measure RNA expression level of RNA sequencing results that has been normalized by both gene length and total reads. RPKM was log transformed and the baseline level (expression status of Agrin−/AChR Ab− group) was normalized to 0 during the data input. The K-means clustering algorithm was used to cluster genes with similar expression pattern. This algorithm calculates the minimum of the following formula:

$$\mathop \sum \limits_{i = 1}^{K} \mathop \sum \limits_{{x_{j} \in S_{i} }} \mathop \sum \limits_{m = 1}^{T} (x_{jm} - c_{im} )^{2}$$

where *K* refers to number of clusters; *S*_1_*, S*_2_,…*, S*_*K*_ refer to each specific cluster; each cluster *S*_*i*_ has a center *c*_*i*_ which represents the mean of the genes *x*_1_,* x*_2_, …, *x*_*j*_ assigned to this cluster_*i*_; and *T* refer to *Group number – *1 (here T is 2). Ten clusters by default was clustered, and the algorithm was performed for 20 series, each time with a different random start. The best performance was included for the final clustering.

### Long non-coding RNA (lncRNA)

The human genome (Ensemble, GRCh38.84) that we applied includes more than 7,000 identified lncRNAs. The HISAT2-HTseq-EdgeR work-flow was used to analyze differentially expressed lncRNAs together with protein-coding RNAs.

### Co-expression network between mRNA and lncRNA

The DE mRNAs and lncRNAs were included to build a mRNA and lncRNA co-expression network. The Pearson correlation coefficient was calculated for all mRNA and lncRNA pairs. Only mRNA-lncRNA pairs with coefficient > 0.9 (positive correlation) or < − 0.9 (negative correlation) was considered as correlated pairs. The mRNA-lncRNA correlated pairs were imported into Cytoscape to make a co-expression network^43^.

### RT-qPCR

RNA sequencing results were confirmed using RT-qPCR and included 6 mRNAs and 1 lncRNA. cDNA was synthesised using SuperScript IV First-Strand Synthesis Kit (Invitrogen). Oligo(dT) primers were used during the cDNA synthesis process. The cDNA product was diluted 1:2 using diethylpyrocarbonate (DEPC)-treated water before introduced into the PCR system. The PCR amplification system (20 μl) included cDNA templates (2 μl), forward primer (1 μl), reverse primer (1 μl), PowerUp SYBR Green Master Mix (Applied Biosystems, Thermo Fisher Scientific, US) (10 μl) and DEPC-treated water (6 μl). The PCR amplification was conducted using Applied Biosystems 7500 Fast Real-Time PCR system Mix (Applied Biosystems). The PCR reaction was started at 50 °C 2 min (UDG activation), and 95 °C 2 min (Dual lock DNA polymerase enzyme), followed by 40 cycles of denature (95 °C °C for 15 s) and annealing/extend (60 °C for 1 min).

The relative standard curve method was used to calculate the expression level of target genes relative to internal reference genes. cDNA templates diluted 1:2, 1:20, 1:200, 1:2,000 and 1:20,000 were used to build the standard curve. For some target genes with low expression levels, dilutions of 1:2, 1:20, 1:200, 1:1,000 and 1:5,000 were used. TUBB and PPIA were used as internal reference genes. Melting curve and agarose gel electrophoresis of PCR products were used to evaluate the specificity of PCR amplification. For PCR product electrophoresis, a mixture of the DNA products (5 μl) and 1 μl 6 × Blue/Orange Loading Dye (Promega, US) was run in 2.5% agarose gel containing 1 μg/ml ethidium bromide for 60 min at 80 V. A 50 bp DNA ladder (Fermentas, US) was used as a standard reference. Information of primers, PCR efficiency, PCR product electrophoresis and a typical standard curve are listed in Fig. S3.

## Supplementary information

Supplementary information
