# Supplementary material for: AChR antibodies show a complex interaction with human skeletal muscle cells in a transcriptomic study
Source: Sci Rep. 2020 Jul 8;10:11230. doi: 10.1038/s41598-020-68185-x (PMC7343820; doi:10.1038/s41598-020-68185-x)
Supplement: Supplementary file 1 — Supplementary information [file 41598_2020_68185_MOESM1_ESM.pdf]

Supplementary Material

AChR antibodies show a complex interaction with human skeletal muscle cells in a transcriptomic study

Yu Hong[1], Xiao Liang[2], Nils Erik Gilhus[1,2]

1. Department of Clinical Medicine, University of Bergen, Bergen, Norway
2. Department of Neurology, Haukeland University Hospital, Bergen, Norway

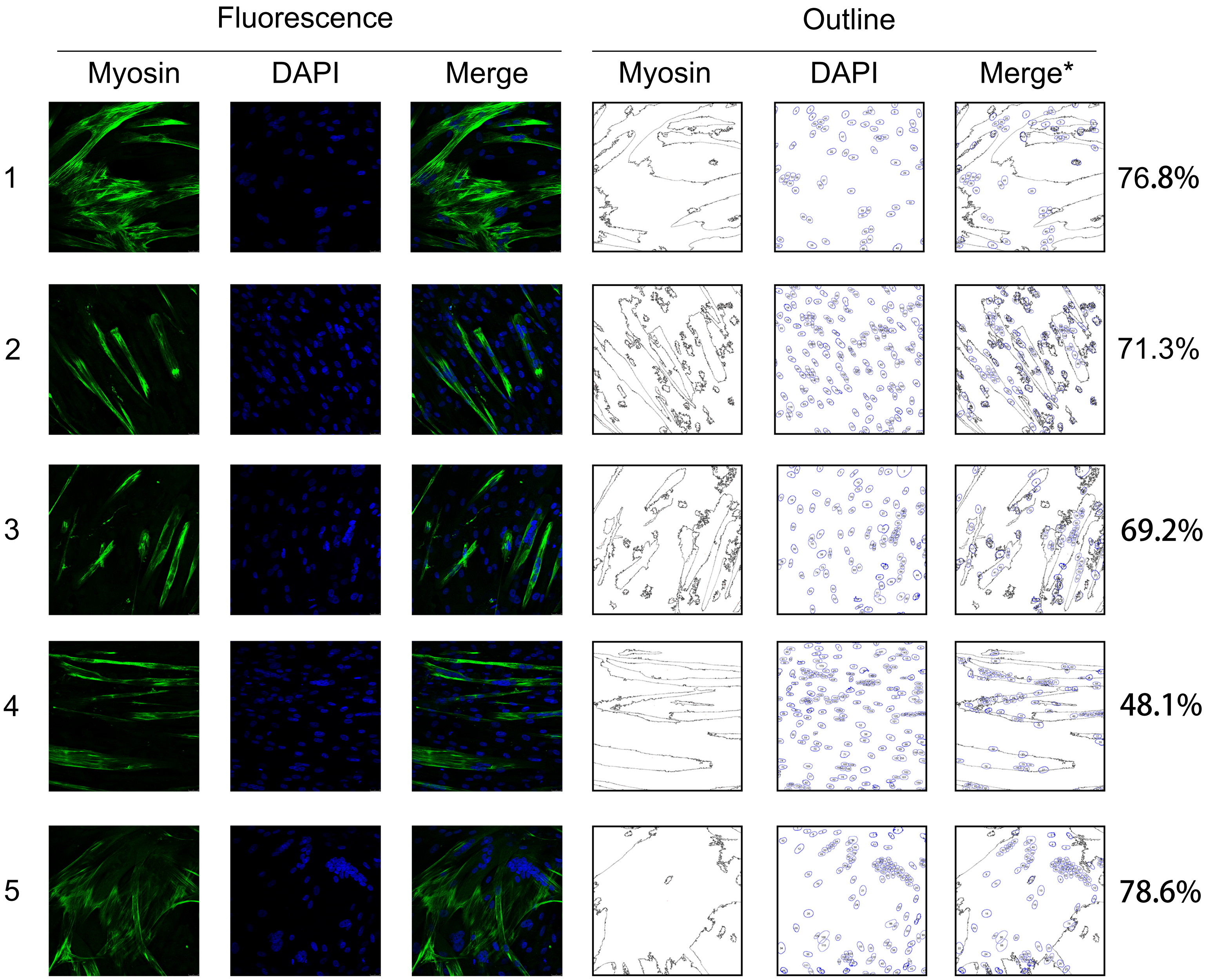

Figure S1. Differentiation efficiency of human myotubes

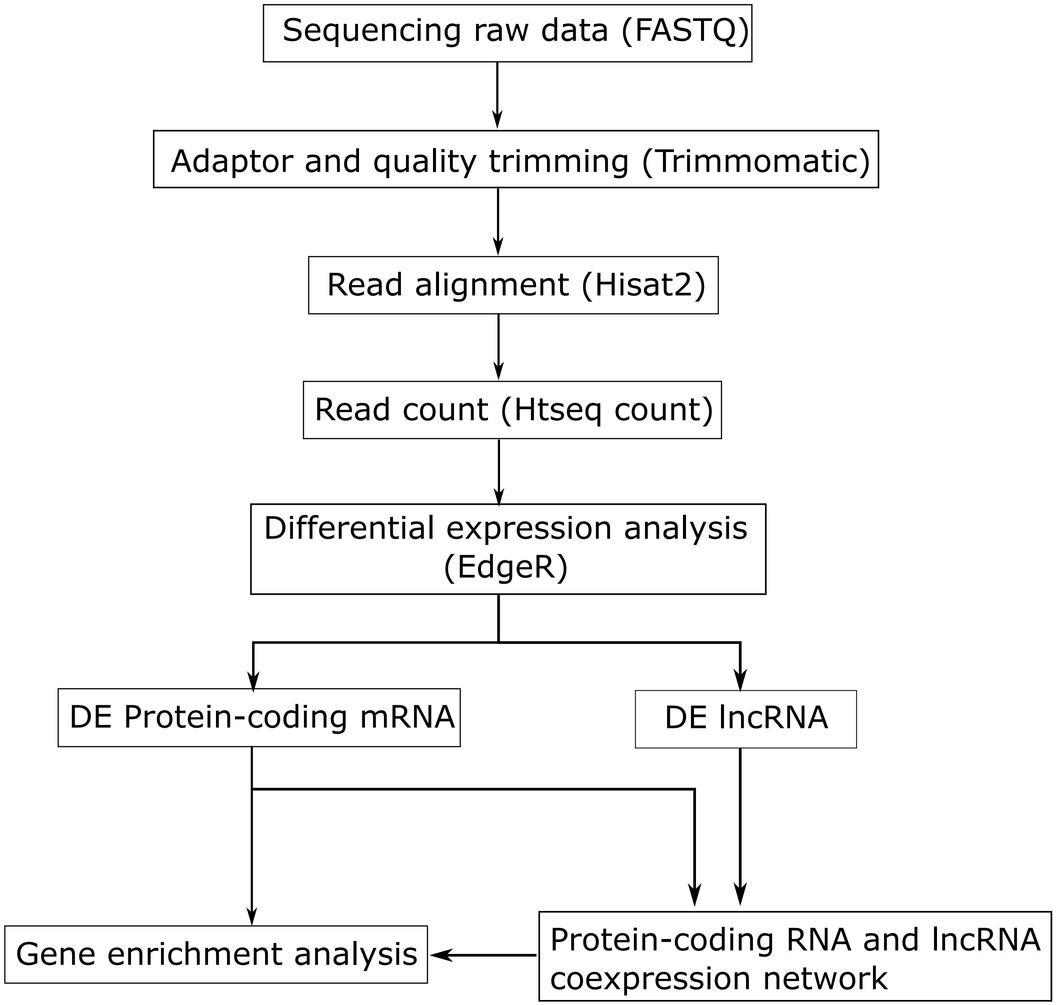

Figure S2. RNA sequencing analysis work-flow

A

Gene	Primer	Product length	Amplification efficiency (%)	Single Tm peak
NR1D2	Forward: 5'-TCAATGGGAGCAGGGGATCTG-3'<br>Reverse:5'-AAAGCCTCCACAGAGTTGACG-3'	158	111.1	Yes
MEG3	Forward: 5'-GGGAAGGGACCTCGAATGTG-3'<br>Reverse:5'-GGGAATAGGTGCAGGGTGTC-3'	85	106	Yes
ARNTL	Forward: 5'-GGTGAGAACCCCCACATAGG -3'<br>Reverse:5'-GAGGCTCATGATGACAGCCA-3'	99	102.4	Yes
ACTC1	Forward: 5'-CCGTACCACAGGCATTGTTC -3'<br>Reverse:5'-GACAAAGGAGTAGCCACGCT-3'	166	97.4	Yes
ACTG1	Forward: 5'-CGCATCCTCCTCTTCTCTGGA -3'<br>Reverse:5'-GCCGCAAGATTCCATACCCAG-3'	130	100.9	Yes
ACTA2	Forward: 5'-CAATGAGCTTCGTGTTGCCC -3'<br>Reverse:5'-GTCATTTTCTCCCGGTTGGC-3'	87	102.9	Yes
PER3	Forward: 5'-AGCAGCCCCAGTTTTCTCAT -3'<br>Reverse:5'-GTGACACAGGCTTGAATGTCG-3'	106	108.3	Yes
TUBB	Forward: 5'-CAACTTTGTATTTGGTCAGTCTGGG -3'<br>Reverse:5'-TAAGGAGAGTGCCCATTCCAG-3'	191	101	Yes

B

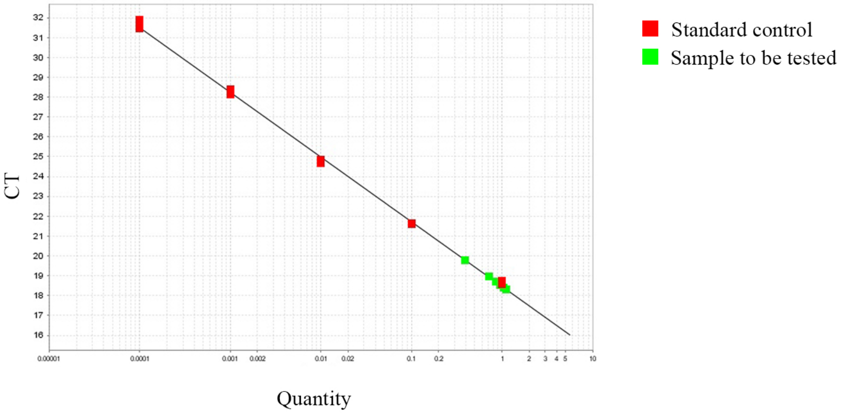

C

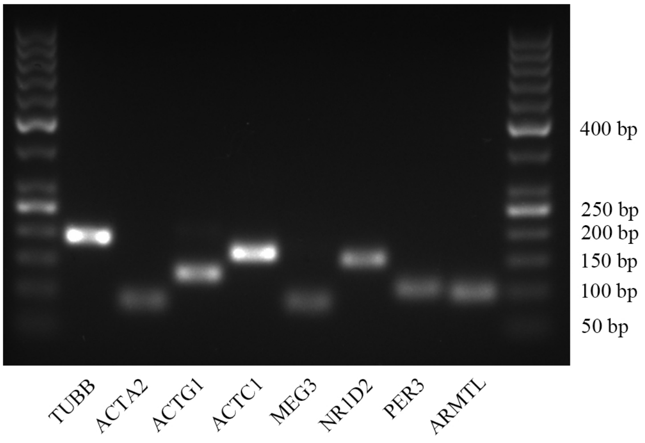

Figure S3. Primers, PCR products and a standard curve for gene expression calculation

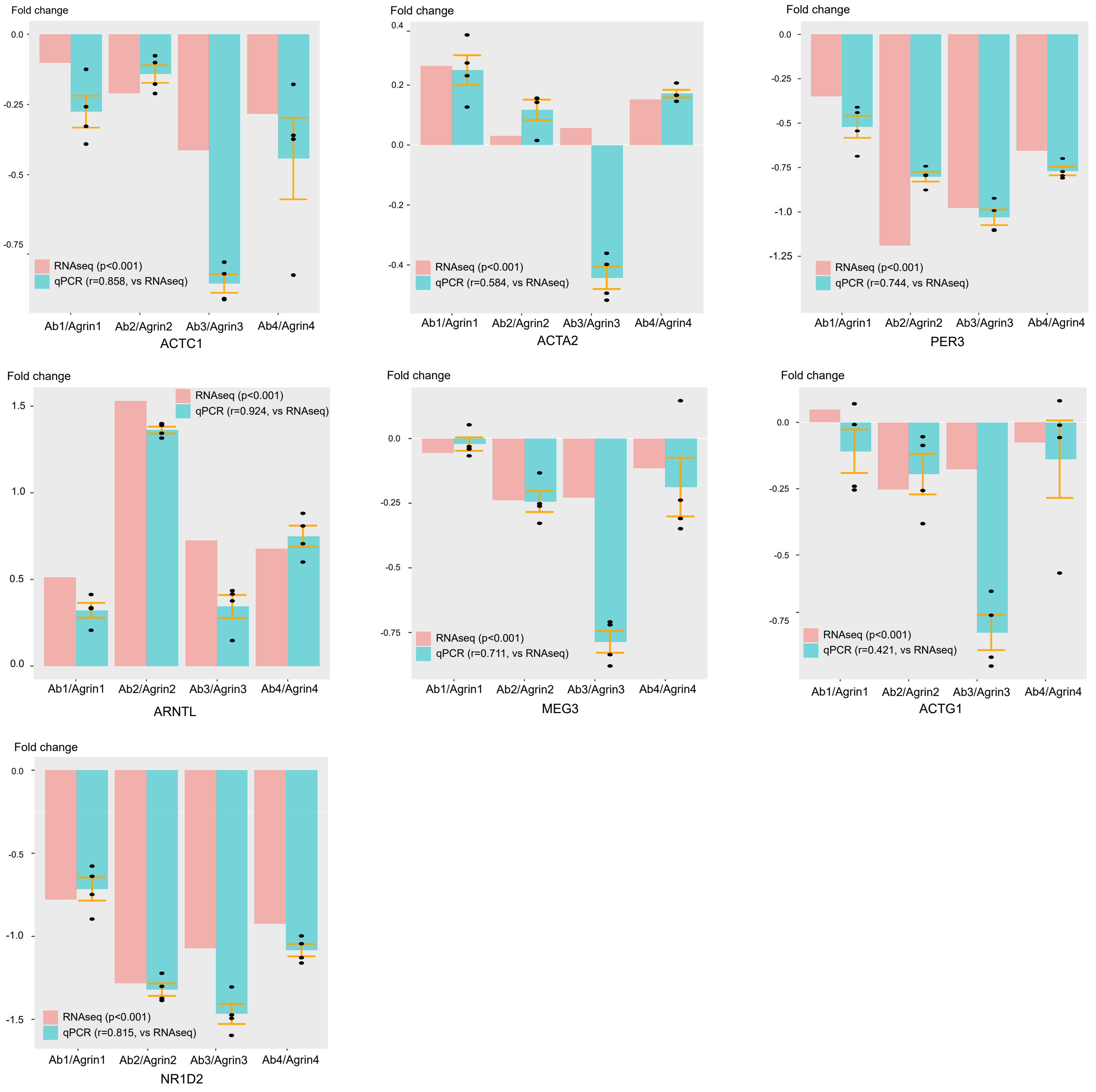

Figure S4. Sample to sample comparsion of RNA sequencing and qRT-PCR results for validated genes

gene_ID	chr	gene_names	gene_length	logFC	logCPM	F	P Value	FDR
ENSG00000106366	7	SERPINE1	3190	-0.58	11.86	538.29	6.43E-119	2.11E-114
ENSG00000174738	3	NR1D2	5820	-1.03	5.78	292.83	1.34E-65	2.20E-61
ENSG00000163661	3	PTX3	1940	-0.36	10.33	138.30	6.42E-32	4.51E-28
ENSG00000159200	21	RCAN1	6990	-0.44	8.81	138.16	6.88E-32	4.51E-28
ENSG00000167074	22	TEF	4715	-0.97	4.30	133.44	7.43E-31	4.06E-27
ENSG00000100234	22	TIMP3	4603	-0.43	8.82	132.46	1.21E-30	5.68E-27
ENSG00000126368	17	NR1D1	2772	-1.30	3.23	125.75	3.56E-29	1.46E-25
ENSG00000049246	1	PER3	8099	-0.82	4.16	89.93	2.50E-21	9.11E-18
ENSG00000179094	17	PER1	7002	-0.91	3.66	84.74	3.43E-20	1.13E-16
ENSG00000104368	8	PLAT	6618	-0.40	7.66	83.18	7.57E-20	2.26E-16
ENSG00000133794	11	ARNTL	6726	0.87	3.53	81.45	1.81E-19	4.95E-16
ENSG00000118523	6	CTGF	2339	-0.28	10.11	79.55	4.75E-19	1.20E-15
ENSG00000154545	X	MAGED4	4809	1.40	2.23	71.13	3.37E-17	7.37E-14
ENSG00000101255	20	TRIB3	3434	-0.40	7.00	70.23	5.32E-17	1.09E-13
ENSG00000274276	21	CBSL	4004	-1.36	1.74	68.69	1.16E-16	2.24E-13
ENSG00000138685	4	FGF2	6775	-0.34	7.86	65.10	7.17E-16	1.31E-12
ENSG00000115419	2	GLS	9827	-0.28	9.49	64.80	8.32E-16	1.44E-12
ENSG00000160201	21	U2AF1	11112	-0.90	2.87	59.74	1.09E-14	1.78E-11
ENSG00000125740	19	FOSB	5553	-0.88	3.49	57.13	4.10E-14	6.40E-11
ENSG00000026559	20	KCNG1	5437	-0.46	5.41	55.15	1.12E-13	1.60E-10
ENSG00000070669	7	ASNS	4455	-0.32	7.70	54.89	1.28E-13	1.75E-10
ENSG00000183160	12	TMEM119	3189	0.30	7.82	52.00	5.58E-13	7.32E-10
ENSG00000059804	12	SLC2A3	6159	-0.37	6.39	50.58	1.15E-12	1.45E-09
ENSG00000205609	16	EIF3CL	3091	0.69	3.62	49.89	1.63E-12	1.98E-09
ENSG00000172432	6	GTPBP2	4624	-0.34	6.89	49.74	1.76E-12	2.04E-09
ENSG00000123358	12	NR4A1	7295	-1.10	2.13	49.62	1.87E-12	2.04E-09
ENSG00000134107	3	BHLHE40	3837	-0.35	6.64	48.24	3.79E-12	4.01E-09
ENSG00000123384	12	LRP1	20839	0.19	11.19	47.30	6.11E-12	6.26E-09
ENSG00000159251	15	ACTC1	4639	-0.25	8.81	45.58	1.47E-11	1.42E-08
ENSG00000147162	X	OGT	9812	-0.25	8.76	44.56	2.47E-11	2.31E-08

Table S1. Differentially expressed protein-coding RNAs. The analysis was conducted to compare Agrin+/Ab- and Agrin+/Ab+ groups. Top 30 differentially expressed genes are listed. CPM: count per million reads. Chr: chromosome. FC: fold change.

gene_ID	chr	gene_names	gene_length	gene_type	logFC	logCPM	F	P Value	FDR
ENSG00000130600	11	H19	2825	lncRNA	-0.86	5.64	198.08	5.77E-45	6.31E-41
ENSG00000272734	10	ADIRF-AS1	5748	antisense	-0.65	2.77	26.94	2.11E-07	6.46E-05
ENSG00000276171	1	AC114498.1	63	lncRNA	0.94	1.10	24.40	7.84E-07	0.00021
ENSG00000248187	4	RP11-184M15.1	1623	lncRNA	-0.36	4.53	21.54	3.46E-06	0.00076
ENSG00000214548	14	MEG3	15769	lncRNA	-0.16	9.20	20.14	7.19E-06	0.0014
ENSG00000247095	11	MIR210HG	2321	lncRNA	-0.38	3.98	19.00	1.31E-05	0.0023
ENSG00000281181	21	CH507-513H4.3	923	lncRNA	-0.32	4.46	17.26	3.26E-05	0.0048
ENSG00000242125	1	SNHG3	4088	lncRNA	-0.29	4.84	17.23	3.31E-05	0.0048
ENSG00000278621	15	CTD-2033D15.3	526	antisense	-0.44	2.85	13.09	0.00030	0.026
ENSG00000260604	6	RP1-140K8.5	7060	lncRNA	-0.19	6.13	12.67	0.00037	0.031
ENSG00000233117	10	LINC00702	6785	lncRNA	-0.42	2.73	11.92	0.00056	0.043
ENSG00000223745	1	CCDC18-AS1	4786	antisense	-0.29	4.13	11.66	0.00064	0.048

Table S2. Differentially expressed lncRNA and anti-sense RNAs. The analysis was conducted to compare Agrin+/Ab- and Agrin+/Ab+ groups. CPM: count per million reads. Chr: chromosome. FC: fold change.
